# Temporal analysis and factors associated with visceral leishmaniasis-HIV-AIDS coinfection in an endemic region of Brazil

**DOI:** 10.1371/journal.pntd.0013845

**Published:** 2025-12-26

**Authors:** Ivair Moura de Souza, Everton Falcão de Oliveira, Kely Cristina Garcia Vilena, Rosilene Rocha Palasson, Gabrielly Segatto Brito, Felipe Machado Mota, Tailma Silva Lino de Souza, Elen Ferraz Teston

**Affiliations:** 1 Postgraduate Program in Nursing, Federal University of Mato Grosso do Sul, Campo Grande, Mato Grosso do Sul, Brazil,; 2 Integrated Institute of Health, Federal University of Mato Grosso do Sul, Campo Grande, Mato Grosso do Sul, Brazil; 3 School of Medicine, Federal University of Mato Grosso do Sul, Campo Grande, Mato Grosso do Sul, Brazil; 4 School of Nursing, Dom Bosco Catholic University, Campo Grande, Mato Grosso do Sul, Brazil; Erasmus MC, University Medical Center Rotterdam, NETHERLANDS, KINGDOM OF THE

## Abstract

**Background:**

The rising incidence of visceral leishmaniasis (VL) and VL/Human Immunodeficiency Virus/ Acquired Immunodeficiency Syndrome (VL-HIV/AIDS) coinfection in parts of Brazil since the early 2000s underscores the need for research to better understand the dynamics of this dual disease burden. Previous analyses from 2000 to 2015 showed increasing trends in occurrence and mortality due to VL/HIV/AIDS. This study aimed to analyze temporal trends in VL and VL/HIV/AIDS coinfection and to identify associated factors.

**Methodology/principal findings:**

We conducted an ecological time-series analysis combined with a cross-sectional study of all confirmed VL cases reported to the Brazilian Notifiable Diseases Information System among residents of Mato Grosso do Sul, Brazil, from 2012 to 2022, including VL-HIV/AIDS coinfections. Temporal trends were evaluated using Joinpoint regression with inflection points identified by the Monte Carlo permutation test. Annual Percentage Change (APC) and Average Annual Percentage Change (AAPC) were estimated with 95% confidence intervals. For the cross-sectional component, VL cases with and without HIV were compared using the chi-square and Mann–Whitney tests. Associated factors were identified through multivariable logistic regression with stepwise selection based on the Akaike Information Criterion. Multicollinearity was assessed using the variance inflation factor, and model fit was evaluated with the Hosmer–Lemeshow test. A significance level of p ≤ 0.05 was adopted. A total of 1,525 confirmed cases of VL were studied, of which 466 (30.55%) presented coinfection. Over the study period, the temporal trend of VL remained stable, while the highest incidence of coinfection occurred in 2022, reaching 1.99 cases per 100,000 inhabitants. Older age, male sex - (adjusted odds ratio [aOR]: 1.58; 95% confidence interval [CI]: 1.19–2.11), and clinical signs such as weight loss (aOR = 2.19; 95% CI: 1.64–2.93) and cough (aOR = 1.89; 95% CI: 1.44–2.49) were significantly associated with a higher likelihood of coinfection. In contrast, symptoms such as fever (aOR=0.28; CI = 0.22–0.37), edema (OR=0.48; CI = 0.36–0.65), splenomegaly (OR=0.52; CI = 0.41–0.65), hepatomegaly (OR=0.62; CI = 0.49–0.78), jaundice (OR=0.62; CI = 0.46–0.84), and other coinfections (OR=0.71; CI = 0.54–0.92) were less frequent among coinfected individuals.

**Conclusions/significance:**

Specific clinical signs and symptoms differ in frequency between coinfected and non-coinfected individuals, which may help identify clinical patterns and improve differential diagnosis strategies. Although temporal trends indicate a stationary pattern over the past 11 years, strengthened interventions are still needed to reduce the incidence of VL–HIV/AIDS coinfection and mitigate its impact in endemic regions. Our findings highlight the importance of early clinical recognition of coinfection patterns and the implementation of targeted public health strategies to mitigate the impact of VL–HIV in endemic regions.

## Author summary

Visceral leishmaniasis is a serious disease caused by the protozoan *Leishmania infantum* or *Leishmania donovani*. If left untreated, it can result in a fatality rate of up to 95%. This situation becomes even more severe when patients are also infected with HIV, with or without progression to AIDS. In Brazil, the number of people affected by both diseases simultaneously—known as visceral leishmaniasis-HIV coinfection—has been increasing. To better understand the factors associated with this condition, we analyzed all cases of visceral leishmaniasis reported in an endemic region, focusing on those with HIV coinfection. Our study included 1,525 confirmed cases of visceral leishmaniasis, of which 466 (30.55%) were coinfected with HIV. We found that being an adult over 40 years old, male, and presenting symptoms such as weight loss significantly increased the likelihood of HIV coinfection among individuals diagnosed with visceral leishmaniasis. These findings highlight the importance of identifying clinical patterns and vulnerable populations to guide public health interventions and improve outcomes for affected patients.

## Introduction

Visceral Leishmaniasis (VL) is a zoonotic disease primarily caused by the protozoan *Leishmania infantum* in the Americas [1]. Among the group of neglected tropical diseases, the condition may reach a fatality rate of up to 95% if not properly treated [2,3]. The World Health Organization estimates that more than 78 countries in tropical and subtropical regions are considered endemic, with about 50,000–90,000 new cases of VL worldwide each year [3–5]. Four countries account for 68% of the global disease burden: India, Sudan, Kenya, and Brazil [1]. In 2019, Brazil accounted for 97% of all reported VL cases in Latin America [6]. Since 1980, Brazil has recorded an increasing number of cases and outbreaks of VL across diverse regions of the country, driven primarily by the adaptation of the vector to urban and peri-urban settings. This ecological shift has significantly contributed to the spatial expansion and dissemination of the disease, posing substantial and ongoing challenges to public health surveillance and control strategies [7,8].

In this context, the expansion of the HIV/AIDS epidemic during the same period contributed to a significant increase in VL-HIV/AIDS coinfection cases, exacerbating the severity of clinical outcomes and therapeutic challenges. [9–12]. In response to this scenario, Brazil has made significant progress in addressing HIV/AIDS. Since the 2000s, improvements in diagnostic methods, including the incorporation of more sensitive rapid tests, have expanded access to testing across health services nationwide, including VL-endemic regions, such as the state of Mato Grosso do Sul. This strengthening of the healthcare network and diagnostic coverage has supported not only the early detection of HIV but also the timely identification of coinfection cases [13].

Additionally, since the implementation of Law No. 9.313/1996, Brazil has guaranteed universal and free access to antiretroviral therapy (ART) through the Unified Health System (SUS), promoting equitable treatment opportunities for people living with HIV [14]. In Mato Grosso do Sul, a state located in the Central-West region of Brazil, these advancements have likely contributed to improved detection of HIV/Leishmania coinfection through earlier diagnosis, as well as better clinical management of both conditions. Temporal and spatial analyses of data from 2000 to 2015 revealed an increasing trend in VL-HIV/AIDS coinfection occurrence and mortality, particularly in the Central-West and Northeast regions [15,16].

Studies on neglected diseases provide critical insights into potential health inequities, particularly in the context of a dual disease burden associated with VL-HIV/AIDS, while simultaneously providing the basis for development of public health policies and the implementation of more effective interventions, while also facilitating the optimal allocation of available resources [17–19]. Projections of case trends and the identification of factors associated with coinfection are essential for guiding clinical management and control strategies. Therefore, the study aimed to analyze the temporal trend of VL cases, VL-HIV/AIDS coinfection, and the associated factors.

## Methods

### Ethics statement

The study was approved by the Federal University of Mato Grosso do Sul Research Ethics Committee (CAAE 67000023.7.0000.0021), and procedures were in accordance with the ethical standards of this committee and with the Helsinki Declaration of 1975, as revised in 1983.

### Study design, setting, and period

The recommendations of the Strengthening the Reporting of Observational Studies in Epidemiology (STROBE) guidelines [20] were followed to organize and structure the presentation of this study.

This study used data from all confirmed VL cases reported to the Notifiable Diseases Information System (SINAN), Brazil’s national epidemiological surveillance tool, among residents of Mato Grosso do Sul, to calculate the time series of annual incidence of VL–HIV/AIDS coinfection from 2012 to 2022.

The system is a tool that monitors health information from the Ministry of Health of Brazil. It is primarily fed by the notification and investigation of cases of diseases and health conditions listed in the national compulsory notification list. These data are made available by the Department of Informatics of the Unified Health System (DataSUS), a platform that centralizes and provides data from various health systems, including SINAN.

According to the Brazilian Institute of Geography and Statistics (IBGE) Mato Grosso do Sul has an estimated population of 2,757,013 in habitants (according to the 2022 census), a territorial area of 357,142.082 km², and a population density of 7.72 in habitants per km². The state borders five other Brazilian states and two South American countries: Paraguay and Bolivia [21].

### Study data

All confirmed VL cases were included, based on the Ministry of Health’s case definition criteria of VL: “They should preferably meet clinical, epidemiological, and laboratory criteria, requiring the identification of the parasite through direct parasitological examination, culture, or immunofluorescence test with a reactive titer of 1:80, combined with the exclusion of other diagnoses, or the performance of a reactive rapid test” [22].

For each case, the following data were extracted from SINAN: sex, age, state and municipality of residence, year of diagnosis, race/skin color, education level, the classic signs and symptoms of VL as recorded in the notification form, comorbidities, HIV coinfection, and case outcome (cure or death). In the present study, the variable related to the occurrence of VL-HIV/AIDS coinfection (yes/no) was defined as the outcome variable in subsequent analyses.

### Data analysis

Although the data were initially obtained at the individual level, we chose to aggregate them and conduct an ecological time-series analysis to examine the variation in the incidence of VL and VL/HIV-AIDS coinfection over the study period. Subsequently, the data were analyzed individually to investigate associations between cases with and without HIV/AIDS coinfection in relation to the clinical and epidemiological variables assessed.

Frequency distribution tables and graphs were generated to describe and characterize the study population. Duplicate notifications, blank records, cases of residents from outside the state, and records in which the presence of HIV or VL–HIV coinfection was marked as “not reported” were excluded from the main analysis. In total, 145 records had this variable marked as “not reported,” while 201 notifications were blank. Additionally, five records referred to residents of other states. The complete database contained 1,876 cases.

#### Analysis of data from the ecological time series study.

The annual incidence of VL and VL-HIV/AIDS coinfection was calculated by dividing the number of new confirmed cases reported each year (numerator) by the estimated total population of Mato Grosso do Sul for the corresponding year (denominator), as provided by the Brazilian Institute of Geography and Statistics (IBGE).

The incidence rate for the period 2012–2022 was determined using the total number of reported cases across the time series as the numerator and the sum of the annual population counts for the period as the denominator, with the result expressed per 100,000 inhabitants.

Temporal trend analyses were conducted with the aim of identifying significant changes over the years studied (2012–2022), covering three distinct outcomes: cases of VL, cases of VL-HIV/AIDS co-infection, and the proportion of VL-HIV/AIDS co-infection cases relative to the total number of VL cases. The analysis was performed using the Joinpoint Regression Program, version 5.3.0, which applies segmented linear regression models with log-linear transformation of the dependent variables.

For the calculation of the first two outcomes, the incidence of cases per 100,000 inhabitants was used to standardize the values relative to the population. For the third outcome, the annual proportion of VL-HIV/AIDS co-infection among VL cases was considered.

To select the number of inflection points and to determine whether models with multiple segments better explained the time series compared to a simple linear model, the Monte Carlo permutation test, with a significance level of 5% (p ≤ 0.05), was employed. Considering the limited number of years available in the time series, the maximum number of joinpoints tested was set at two to avoid overfitting.

The Annual Percentage Change (APC) and the Average Annual Percentage Change (AAPC), both with 95% confidence intervals (95% CI), were calculated to identify whether there was an increasing, decreasing, or stationary trend during the evaluated period, both for the individual segments and for the overall time frame analyzed.

#### Analysis of cross-sectional study data.

Among reported cases of VL, the association between cases with VL-HIV/AIDS coinfection and those without HIV/AIDS coinfection regarding clinical-epidemiological variables was assessed as follows: categorical variables were analyzed using the chi-square test, while numerical variables were compared between groups (with and without coinfection) using the Mann-Whitney U test due to the non-normal distribution of the data. Adjusted odds ratios (aOR) were obtained through multivariable logistic regression, with 95% confidence intervals (95% CI), to estimate the strength of the association between each variable and the presence of VL-HIV/AIDS coinfection.

For the multivariable analysis, covariates with a p-value ≤ 0.10 in the univariate analysis were selected. A binomial logistic regression model was used in this phase of the analysis. Variable selection, adjustment for potential confounders, and model selection were conducted using a stepwise algorithm (both backward and forward directions) based on the Akaike Information Criterion (AIC).

Multicollinearity was assessed using the Variance Inflation Factor (VIF), considering values above 5 as indicative of potential multicollinearity problems. Variables with VIF > 5 were carefully examined and considered for removal or adjustment to improve model stability. The overall model fit was evaluated using the Hosmer-Lemeshow goodness-of-fit test. A significance level of p ≤ 0.05 was adopted for all hypothesis tests.

## Results

### Ecological study of time series

**Fig 1:**
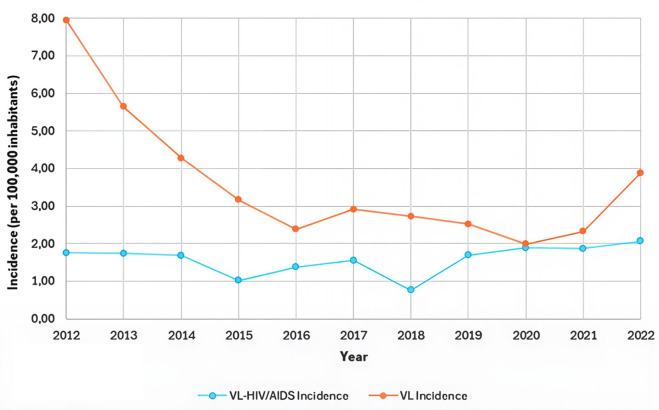
Temporal evolution of VL incidence with and without VL-HIV/AIDS coinfection in Mato Grosso do Sul (2012-2022) Brazil.

A total of 1,525 confirmed VL cases were reported in Mato Grosso do Sul during the study period, 466 of which VL-HIV/AIDS coinfection cases. Fig 1 illustrates the temporal distribution of these cases over the 11 years analyzed.

The highest VL incidence occurred in 2012 (7.98 per 100,000 inhabitants), while the lowest was in 2020 (1.99 per 100,000). For VL-HIV/AIDS coinfection, the lowest incidence rates were in 2018 (0.76 per 100,000), whereas 2022 recorded the highest incidence (2.07 per 100,000) (Fig 1). Over the entire study period (2012–2022), the incidence rate of VL was 3.56 new cases per 100,000 inhabitants per year. For VL–HIV/AIDS coinfection, the incidence rate was 1.58 cases per 100,000 inhabitants per year.

Considering that 145 donation records had the HIV diagnosis marked as “not informed” and 201 were left blank, a sensitivity analysis was conducted to assess the impact of the uncertainty generated by these missing data in the SINAN system on the incidence rate of coinfection. In addition to the scenario used in the study, in which cases with missing data were excluded (reduced dataset: n = 1,525), two additional scenarios were simulated to estimate the potential effect of the missing information on the results.

For this purpose, the dataset was used, excluding only records from other states, without removing cases with missing information (VL cases – n = 1,871; VL-HIV/AIDS coinfections – n = 466; blank or “not informed” records – n = 346). The first and most pessimistic scenario applied a proportional extrapolation, assuming that the coinfection rate among incomplete records was equivalent to that observed among cases with complete data (18.49%), resulting in approximately 530 coinfected cases (466 + 346 × 0.1849).

The second, intermediate scenario adopted a conservative estimate, attributing coinfection to 10% of the 346 records with missing information, totaling approximately 500 cases (466 + 346 × 0.10). Based on these projections, the estimated incidence rate per 100,000 inhabitants per year was 1.58 in the study’s baseline scenario, which excluded missing data, 1.68 in the intermediate scenario, and 1.79 in the pessimistic scenario.

The time series analysis indicated a statistically significant decreasing trend in the incidence of VL cases without VL-HIV/AIDS coinfection between 2012 and 2016. During this period, the APC was -27.72% per year (95% Confidence Interval (CI): -37.49; -11.73). Between 2016 and 2022, an increase in incidence was observed, with an APC of 4.38% per year (95% CI: -6.67; 16.76), although this growth was not statistically significant, indicating a stable trend.

Regarding VL-HIV/AIDS coinfection cases, a decreasing trend was identified between 2012 and 2018, with an average annual reduction of -5.50% (95% CI: -24.26; 1.72). Starting in 2018, there was an inflection point in the trend, with a significant increase in incidence between 2018 and 2022, showing an APC of 15.51% per year (95% CI: 3.07; 45.06).

**Fig 2:**
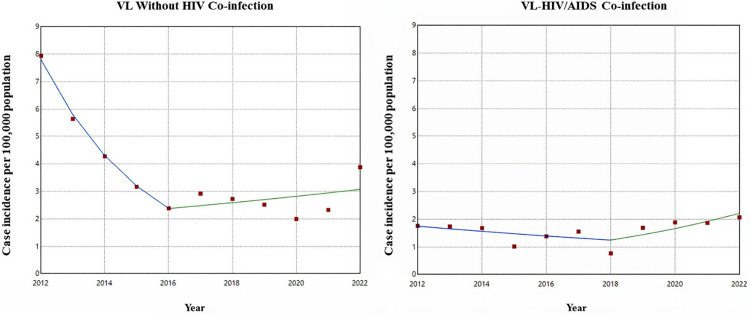
Time series analysis for the incidence of visceral leishmaniasis cases with and without HIV in Mato Grosso do Sul, Brazil. 2012-2022.

Considering the entire analyzed period, the Average Annual Percentage Change (AAPC) for VL cases was -8.89% (95% CI: -11.68; -6.22), indicating a decreasing trend over time. For VL-HIV/AIDS coinfection cases, the AAPC was 2.39% (95% CI: -1.88; 6.52), suggesting that, despite the significant reduction observed up to 2018, the overall trend during the studied period can be considered stationary (Fig 2).

**Fig 3:**
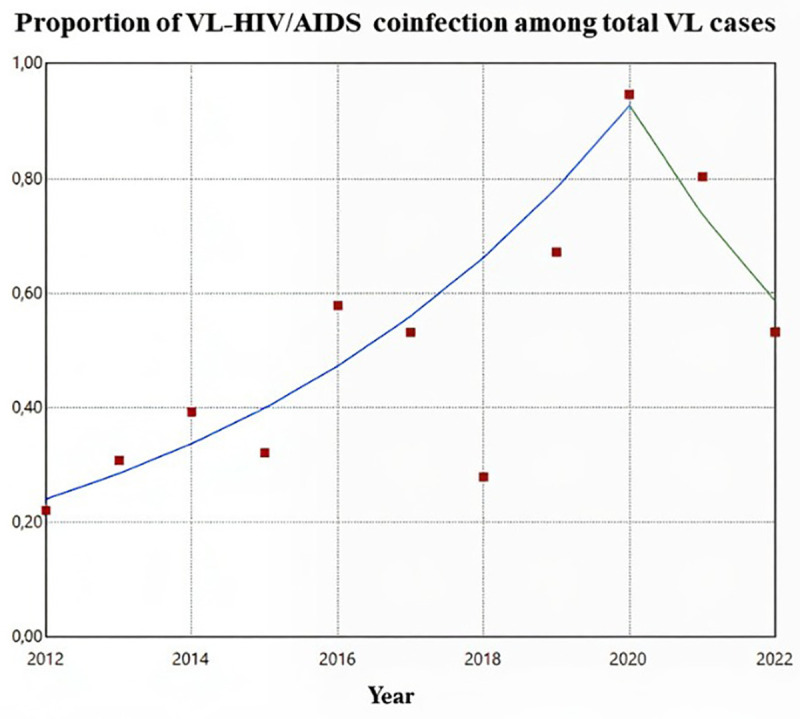
Time series analysis for the proportion of VL-HIV/AIDS coinfection among total VL cases in Mato Grosso do Sul, Brazil. 2012-2022.

When analyzing the trend in the proportion of VL-HIV/AIDS coinfection cases relative to the total number of VL cases, a significant increase was observed between 2012 and 2020, with an APC of 18.30% (95% CI: 15.38; 31.24). From 2020 onwards, there was an inflection in the trend, with a mean reduction of 20.50% per year (95% CI: -39.41; -4.96). For the entire analyzed period, the Average Annual Percentage Change (AAPC) was statistically significant, indicating a growth of 9.25% (95% CI: 4.41; 17.07) (Fig 3).

### Cross-sectional study

The overall median age was 37 years (IQR: 15–51). Individuals with VL-HIV/AIDS coinfection had a significantly higher median age compared to those without coinfection (41 years [IQR: 34–48] vs. 30 years [IQR: 6–54]; p < 0.001) (Table 1).

**Table 1:** Characterization and Frequency distribution of participants with and without VL-HIV/AIDS coinfection according to clinical-epidemiological profile in Mato Grosso do Sul (2012 to 2022). Brazil, 2024.

	Total (n = 1525)	VL (n = 1059)	VL-HIV/Aids (n = 466)	p-value*	OR (95% CI)	aOR (95% CI)
Age (average, SD)	34.8 (23.4)	31.8 (26.4)	41.6 (11.8)	<0.001	1.02 (1.01;1.02)	1.01 (1.00;1.02)
	**n (%)**	**n (%)**	**n (%)**			
**Sex**						
Female	522 (34.3)	408 (38.5)	114 (24.5)	Ref.	Reference	Reference
Male	1002 (65.7)	651 (61.5)	351 (75.5)	<0.001	1.93 (1.51;2.47)	1.58 (1.19;2.11)
**Color/Race**						
White	380 (27.1)	258 (26.5)	122 (28.3)	Ref.	Reference	–
Non-white	1024 (72.9)	715 (73.5)	309 (71.7)	0.486	0.91 (0.71;1.18)	–
**Education**						
Non-literate	27 (3.7)	18 (3.8)	9 (3.5)	Ref.	Reference	–
Basic education	455 (62.6)	298 (63.3)	157 (61.3)	0.918	1.04 (0.47;2.51)	–
High school	198 (27.2)	128 (27.2)	70 (27.3)	0.852	1.08 (0.47;2.68)	–
Higher education	47 (6.5)	27 (5.7)	20 (7.81)	0.450	1.47 (0.55;4.10)	–
**Fever**						
No	283 (18.9)	131 (12.5)	152 (33.6)	Ref.	Reference	Reference
Yes	1217 (81.1)	917 (87.5)	300 (66.4)	0.000	0.28 (0.22;0.37)	0.31 (0.22;0.42)
**Weakness**						
No	295 (20.0)	215 (20.9)	80 (17.8)	Ref.	Reference	–
Yes	1181 (80.0)	812 (79.1)	369 (82.2)	0.168	1.22 (0.92;1.63)	–
**Edema**						
No	1076 (76.0)	716 (72.4)	360 (84.5)	Ref.	Reference	Reference
Yes	339 (24.0)	273 (27.6)	66 (15.5)	<0.001	0.48 (0.36;0.65)	0.54 (0.38;0.76)
**Weigth loss**						
No	508 (34.7)	396 (39.0)	112 (24.8)	Ref.	Reference	Reference
Yes	958 (65.3)	619 (61.0)	339 (75.2)	<0.001	1.93 (1.51;2.49)	2.19 (1.64;2.93)
**Cough**						
No	810 (56.3)	582 (57.9)	228 (52.5)	Ref.	Reference	Reference
Yes	629 (43.7)	423 (42.1)	206 (47.5)	0.060	1.24 (0.99;1.56)	1.89 (1.44;2.49)
**Pallor**						
No	523 (36.1)	376 (37.2)	147 (33.8)	Ref.	Reference	–
Yes	924 (63.9)	636 (62.8)	288 (66.2)	0.223	1.16 (0.92;1.47)	–
**Splenomegaly**						
No	566 (39.0)	346 (34.1)	220 (50.1)	Ref.	Reference	Reference
Yes	887 (61.0)	668 (65.9)	219 (49.9)	<0.001	0.52 (0.41;0.65)	0.73 (0.53;1.00)
**Hemorrhage**						
No	1251 (88.8)	878 (89.5)	373 (87.4)	Ref.	Reference	–
Yes	157 (11.2)	103 (10.5)	54 (12.6)	0.243	1.24 (0.86;1.75)	–
**Hepatomegaly**						
No	571 (39.4)	363 (35.9)	208 (47.5)	Ref.	Reference	Reference
Yes	878 (60.6)	648 (64.1)	230 (52.5)	<0.001	0.62 (0.49;0.78)	0.82 (0.60;1.14)
**Jaundice**						
No	1116 (79.3)	759 (77.1)	357 (84.4)	Ref.	Reference	Reference
Yes	292 (20.7)	226 (22.9)	66 (15.6)	0.002	0.62 (0.46;0.84)	0.63 (0.44;0.90)
**Other co-infections**						
No	983 (70.9)	665 (68.8)	318 (75.7)	Ref.	Reference	–
Yes	404 (29.1)	302 (31.2)	102 (24.3)	0.008	0.71 (0.54;0.92)	–
**Death by VL**						
No	1349 (92.9)	927 (92.8)	422 (93.2)	Ref.	Reference	–
Yes	103 (7.09)	72 (7.21)	31 (6.84)	0.812	0.95 (0.60;1.46)	–

Notice: *Chi-square or the Mann-Whitney test; VL = Visceral leishmaniasis; OR= Odds ratio; aOR= Odds ratio adjusted.

When analyzing signs and symptoms potentially associated with VL-HIV/AIDS coinfection (Table 1), which could aid in the suspicion of coinfection diagnosis, it was found that weight loss was more common among individuals with coinfection (aOR: 2.19, 95% CI: 1.64–2.93), as was cough (aOR: 1.89, 95% CI: 1.44–2.49). Some signs and symptoms were less frequent in individuals with coinfection, such as fever (aOR = 0.31, 95% CI: 0.22–0.42), edema (aOR = 0.54, 95% CI: 0.38–0.76), splenomegaly (OR=0.52; CI = 0.41–0.65), hepatomegaly (OR=0.62; CI = 0.49–0.78), and jaundice (aOR = 0.63, 95% CI: 0.44–0.90).

## Discussion

The time series analysis indicated a statistically significant decreasing trend in the incidence of VL cases between 2012 and 2016, and a significant increase in the incidence of VL/HIV/AIDS coinfection between 2018 and 2022. It was identified that some signs and symptoms may be more common or sporadic in coinfected individuals, which may aid in case surveillance and differential diagnosis. The data show that the pattern of VL endemicity observed at the state level in Mato Grosso do Sul since the early 2000s [23] has persisted, without individual analysis of specific municipalities. It should also be considered that the number of cases may be even higher due to underreporting and barriers to accessing healthcare services, particularly specialized services [24,25].

The increase in the incidence of VL/HIV/AIDS coinfection beginning in 2018 may reflect a combination of social, epidemiological, and operational factors. The persistence of VL in vulnerable urban areas, the expansion of populations at risk for HIV, and gaps in systematic HIV testing among VL cases likely contribute to this pattern, as individuals with VL who are not routinely screened for HIV often have their coinfection initially missed. Additional factors include the limited coordination between disease control programs, delays in HIV diagnosis, and barriers to accessing specialized care [26–28]. Patients who receive an HIV diagnosis only at advanced stages commonly present pronounced immunosuppression, making them more susceptible to Leishmania infantum infection; this delay can therefore lead to an increased number of VL cases among people living with HIV/AIDS, amplifying the observed rise in coinfection. Moreover, the increasing trend in HIV/AIDS cases in the study region between 2009 and 2018 may also have contributed to the rise in the incidence rate of the coinfection [29].

Studies suggest that the frequency of VL and VL–HIV/AIDS coinfection in the male population may be related to occupational activities that increase exposure to risk areas, in addition to hormonal factors that may influence the immune response to the parasite [26,30]. Highlight that, in endemic areas of Northeastern Brazil, the male predominance among coinfection cases is also associated with sociodemographic factors, such as greater mobility for work and increased social vulnerability [31]. Furthermore, economic and structural changes in the region, such as the expansion of construction projects and enterprises that attract labor from different locations, have intensified migratory flows, increasing the movement of susceptible individuals and facilitating the spread of the disease among previously unexposed population groups [26,32,33]. When this is combined with the presence of comorbidities, the propensity for opportunistic infections increases in individuals diagnosed with HIV [34].

In addition, low adherence to antiretroviral therapy and adequate care are hypotheses that may explain the high frequency of coinfection [26]. Thus, the prevention of opportunistic infections in individuals infected with HIV, together with the investigation of suspected cases, animal protection measures, such as the use of collars impregnated with deltamethrin and laboratory monitoring (including LT-CD4 + count and HIV viral load testing) from the beginning of follow-up, in addition to the investigation of comorbidities and coinfections, constitute effective interventions [15,33,35].

VL-HIV/AIDS coinfection presents complex challenges in the treatment and management of patients, since both diseases can share symptoms, which complicates diagnosis. HIV compromises the immune system, making VL more severe and aggressive, and can lead to an atypical presentation of the disease [36]. Thus, monitoring clinical manifestations becomes essential to assess severity, adjust treatment, identify complications, and manage side effects of medications.

However, the presence of atypical manifestations in cases of VL-HIV/AIDS coinfection, including disseminated cutaneous lesions and resistance to treatment, has been reported [37]. Similarly, another study on coinfection highlighted that although the clinical presentation of coinfected cases is similar to that observed in patients not infected with HIV, coinfection can lead to a more severe clinical course, with atypical signs and symptoms and resistance to treatment [38].

In light of these findings, frequent training for healthcare professionals is essential to enable them to recognize atypical signs as indicators of coinfection, rather than isolated symptoms of a single disease. This approach facilitates the early diagnosis of VL in patients with HIV/AIDS. Additionally, differentiated therapeutic protocols for individuals with coinfection can contribute to reducing mortality and associated complications.

The occurrence of weight loss associated with greater chances of VL-HIV/AIDS coinfection may be related to interactions between socioeconomic conditions, health, and weight loss. In groups with low socioeconomic status, access to nutritious food and health care is often limited, which can result in a higher prevalence of diseases, such as VL-HIV/AIDS coinfection, in addition to difficulties in maintaining a healthy weight. Furthermore, late diagnosis of coinfection and malnutrition can worsen weight loss [39].

The low odds of occurrence of fever in cases of VL-HIV/AIDS coinfection can be attributed to several reasons related to the interaction between the two infections and the compromised immune response caused by HIV. This virus negatively affects the immune system, especially CD4 + T cells, which are essential for the inflammatory response and defense against infections. The decrease in these cells can reduce the body’s ability to generate an adequate inflammatory response, including the manifestation of fever [40]. Furthermore, VL can manifest itself in different ways depending on the individual’s immune status and the intensity of the infection. The absence of specific symptoms, such as weakness, edema, hemorrhage, jaundice, and splenomegaly, as well as overlap with other infections, can make early diagnosis of both VL and VL-HIV/AIDS coinfection difficult [41,42].

It is believed that the use of secondary data may have contributed to underreporting and, consequently, underestimation of the actual number of VL-HIV/AIDS coinfection cases. Moreover, the database used was not originally designed for scientific research purposes, which limited the collection of more detailed information—particularly regarding the longitudinal follow-up of individuals.

It is important to note that, because our analysis is restricted to deaths attributed specifically to visceral leishmaniasis, the estimated case-fatality rate is based solely on this outcome and does not account for deaths from other causes. Thus, the absence of a difference in LV-attributed deaths between individuals with and without coinfection may be related to this limitation of the information system. Consequently, potential differences in overall mortality between LV/HIV+ and LV/HIV– individuals may not have been detected. Furthermore, the reported case-fatality rate reflects only cases with documented outcomes, making it impossible to fully assess mortality among individuals with missing or indeterminate HIV status.

This finding supports the hypothesis of an underestimation of the true incidence of coinfection, which may be higher than reported. Nevertheless, the results of this study provide important insights to inform discussions on clinical patterns and the challenges associated with the differential diagnosis of VL-HIV/AIDS coinfection.

The findings of this study provide support for expanding surveillance efforts to include older individuals and males, particularly in endemic areas. It is also recommended to conduct awareness campaigns targeting these populations about the risk of co-infection, as well as to streamline service workflows to facilitate early diagnosis. Additionally, the significant association between weight loss and cough with a higher likelihood of co-infection suggests that these symptoms should be considered warning signs for more in-depth investigations in HIV patients, especially in endemic areas of leishmaniasis.

## Conclusion

The temporal and associated factors analysis of VL and VL-HIV/AIDS coinfection in Mato Grosso do Sul (2012–2022) revealed a significant decrease in VL incidence, but a notable increase in coinfection cases between 2018 and 2022, indicating an overall stationary trend for the full period. The proportion of coinfection among total VL cases also showed a significant upward trend.

Coinfected individuals were predominantly older males and more frequently presented symptoms such as weight loss and cough. In contrast, classic VL signs—such as fever, splenomegaly, and jaundice—were less common, possibly due to HIV-related immunosuppression.

These findings underscore the importance of recognizing atypical clinical presentations and prioritizing vulnerable groups in surveillance and early diagnosis efforts. Limitations related to the use of secondary data, including underreporting and missing information, may have contributed to an underestimation of the true incidence.

VL-HIV/AIDS coinfection remains a growing public health challenge and calls for integrated strategies for surveillance, prevention, and clinical management to reduce its associated morbidity and mortality in endemic regions.

## References

[1] World Health Organization. Leishmaniasis [Internet]; 2023 [cited 2024 Jan 6]. Available from: https://www.who.int/news-room/fact-sheets/detail/leishmaniasis

[2] Matlashewski G, Arana B, Kroeger A, Battacharya S, Sundar S, Das P, et al. Visceral leishmaniasis: elimination with existing interventions. Lancet Infect Dis. 2011;11(4):322–5. doi: 10.1016/S1473-3099(10)70320-0 21453873

[3] Vieira-Duarte R, Araújo VEM de, Veloso GA, Cardoso DT, Ker FTO, Barbosa DS, et al. Mortality due to visceral leishmaniasis in Brazil by municipalities, 2001-2018: a spatial-temporal analysis of estimates from the Global Burden of Disease study. Public Health. 2024;234:58–63. doi: 10.1016/j.puhe.2024.06.003 38954883

[4] Azevedo TS de, Lorenz C, Chiaravalloti-Neto F. Risk mapping of visceral leishmaniasis in Brazil. Rev Soc Bras Med Trop. 2019;52:e20190240. doi: 10.1590/0037-8682-0240-2019 31778399

[5] World Health Organization. Leishmaniasis: Fact sheet [Internet]; 2019 [cited 2023 Nov 6]. Available from: https://www.who.int/en/news-room/fact-sheets/detail/leishmaniasis

[6] Organización Panamericana de la Salud. Atlas interactivo de leishmaniasis en las Américas: aspectos clínicos y diagnósticos diferenciales [Internet]. Washington (DC): Organización Panamericana de la Salud; 2020 [cited 2025 Aug 11]. Available from: https://iris.paho.org/handle/10665.2/52645

[7] Maia-Elkhoury ANS, Alves WA, Sousa-Gomes ML de, Sena JM de, Luna EA. Visceral leishmaniasis in Brazil: trends and challenges. Cad Saude Publica. 2008;24(12):2941–7. doi: 10.1590/s0102-311x2008001200024 19082286

[8] De Almeida Marzochi MC, et al. Visceral leishmaniasis in Brazil: scenarios and challenges for the surveillance and control. Patol Trop. 2023;52(1):1–10. Available from: https://revistas.ufg.br/iptsp/article/view/74769

[9] Leite de Sousa-Gomes M, Romero GAS, Werneck GL. Visceral leishmaniasis and HIV/AIDS in Brazil: Are we aware enough? PLoS Negl Trop Dis. 2017;11(9):e0005772. doi: 10.1371/journal.pntd.0005772 28945816 PMC5612457

[10] Costa LDLN, Lima US, Rodrigues V, Lima MIS, Silva LA, Ithamar J, et al. Factors associated with relapse and hospital death in patients coinfected with visceral leishmaniasis and HIV: a longitudinal study. BMC Infect Dis. 2023;23(1):141. doi: 10.1186/s12879-023-08009-1 36882732 PMC9993705

[11] De Lima RG, Mendonça TM, Mendes T da S, Menezes MVC. Perfil epidemiológico da leishmaniose visceral no Brasil, no período de 2010 a 2019. Acervo Saúde. 2021;13(4):e6931. doi: 10.25248/reas.e6931.2021

[12] Lindoso JAL, Moreira CHV, Cunha MA, Queiroz IT. Visceral leishmaniasis and HIV coinfection: current perspectives. HIV AIDS (Auckl). 2018;10:193–201. doi: 10.2147/HIV.S143929 30410407 PMC6197215

[13] Maksud I, Fernandes NM, Filgueiras SL. Technologies for HIV prevention and care: challenges for health services. Rev Bras Epidemiol. 2015;18 Suppl 1:104–19. doi: 10.1590/1809-4503201500050008 26630301

[14] Brazil. Law No. 9,313, of November 13, 1996. Provides for the free distribution of medicines to people living with HIV and AIDS patients. Brasília (DF): Diario Oficial da Uniao; 1996;Sect. 1. Portuguese.

[15] Martins-Melo FR, Lima M da S, Alencar CH, Ramos AN Jr, Heukelbach J. Epidemiological patterns of mortality due to visceral leishmaniasis and HIV/AIDS co-infection in Brazil, 2000-2011. Trans R Soc Trop Med Hyg. 2014;108(6):338–47. doi: 10.1093/trstmh/tru050 24706340

[16] Graepp-Fontoura I, Barbosa DS, Fontoura VM, Guerra RNM, Melo S de A, Fernandes MN de F, et al. Visceral leishmaniasis and HIV coinfection in Brazil: epidemiological profile and spatial patterns. Trans R Soc Trop Med Hyg. 2023;117(4):260–70. doi: 10.1093/trstmh/trac093 36219448

[17] Ribeiro CJN, Dos Santos AD, Lima SVMA, da Silva ER, Ribeiro BVS, Duque AM, et al. Space-time risk cluster of visceral leishmaniasis in Brazilian endemic region with high social vulnerability: an ecological time series study. PLoS Negl Trop Dis. 2021;15(1):e0009006. doi: 10.1371/journal.pntd.0009006 33465104 PMC7846114

[18] Werle JE, Teston EF, Rossi RM, Frota OP, Ferreira Júnior MA, Cunha GH da, et al. Fatores associados ao óbito por HIV/Aids. Acta Paul Enferm. 2022;35:eAPE02837. doi: 10.37689/acta-ape/2022ao02837

[19] Souza EA de, Boigny RN, Oliveira HX, Oliveira MLW-D-R de, Heukelbach J, Alencar CH, et al. Tendências e padrões espaço-temporais da mortalidade relacionada à hanseníase no Estado da Bahia, Nordeste do Brasil, 1999-2014. Cad Saúde Colet. 2018;26(2):191–202. doi: 10.1590/1414-462x201800020255

[20] Malta M, Cardoso LO, Bastos FI, Magnanini MMF, Silva CMFP da. STROBE initiative: guidelines on reporting observational studies. Rev Saude Publica. 2010;44(3):559–65. doi: 10.1590/s0034-89102010000300021 20549022

[21] Brazilian Institute of Geography and Statistics. Cities and States: Mato Grosso do Sul [Internet]. IBGE; [cited 2025 Jan 22]. Available from: https://www.ibge.gov.br/cidades-e-estados/ms.html

[22] Brazil. Ministry of Health. Health Surveillance Secretariat. Department of Epidemiological Surveillance. Visceral leishmaniasis: clinical recommendations for reducing lethality [Internet]. Brasília: Ministry of Health; 2011 [cited 2025 Apr 28]. 78 p. (Series A. Standards and Technical Manuals). Available from: https://bvsms.saude.gov.br/bvs/publicacoes/leishmaniose_visceral_reducao_letalidade.pdf

[23] Correa Antonialli SA, Torres TG, Paranhos Filho AC, Tolezano JE. Spatial analysis of American visceral leishmaniasis in Mato Grosso do Sul State, Central Brazil. J Infect. 2007;54(5):509–14. doi: 10.1016/j.jinf.2006.08.004 16979241

[24] Machado CAL, Valle D, Horta MC, Meiga AYY, Sevá A da P. Patterns and drivers of human visceral leishmaniasis in Pernambuco (Brazil) from 2007 to 2018. PLoS Negl Trop Dis. 2023;17(2):e0011108. doi: 10.1371/journal.pntd.0011108 36753511 PMC9983839

[25] Nina LN da S, Caldas A de JM, Soeiro VM da S, Ferreira TF, Silva TC, Rabelo PPC. Spatial-temporal distribution of visceral leishmaniasis in Brazil from 2007 to 2020Distribución espaciotemporal de la leishmaniasis visceral en Brasil en el período 2007-2020. Rev Panam Salud Publica. 2023;47:e160. doi: 10.26633/RPSP.2023.160 38024446 PMC10648444

[26] Chaves AF de CP, Costa IVS, Brito MO de, Sousa Neto FA de, Mascarenhas MDM. Visceral leishmaniasis in Piauí, Brazil, 2007-2019: an ecological time series analysis and spatial distribution of epidemiological and operational indicators. Epidemiol Serv Saude. 2022;31(1):e2021339. doi: 10.1590/S1679-49742022000100013 35588511

[27] Cavalcante KK de S, Borges KMO, Cavalcante FRA, Correia FGS, Florêncio CMGD, Alencar CH. Epidemiological aspects and high magnitude of human visceral leishmaniasis in Ceará, Northeast of Brazil, 2007-2021. Rev Soc Bras Med Trop. 2022;55:e06842021. doi: 10.1590/0037-8682-0684-2021 35613225 PMC9131778

[28] Silva CAS, Serpa PF. The migratory flow in the state of Mato Grosso do Sul: reception of refugees and international immigrants. Rev Metaxy. 2019;2(1):31–55. Available from: https://revistas.ufrj.br/index.php/metaxy/article/view/20425

[29] Werle JE, Teston EF, Marcon SS, Cunha GH da, Mandu JB dos S, Ferreira Junior MA. HIV/AIDS em região de tríplice fronteira: subsídios para reflexões sobre políticas públicas. Esc Anna Nery. 2021;25(3):e20200320. doi: 10.1590/2177-9465-ean-2020-0320

[30] Camargo Júnior RNC, Sarmento Gomes JS, Corrêa Carvalho MC, Chalkidis H de M, da Silva WC, Sousa da Silva J, et al. Visceral leishmaniasis associated with HIV coinfection in Pará, Brazil. HIV AIDS (Auckl). 2023;15:247–55. doi: 10.2147/HIV.S400189 37255531 PMC10226483

[31] Machado CAL, Sevá A da P, Silva AAFAE, Horta MC. Epidemiological profile and lethality of visceral leishmaniasis/human immunodeficiency virus co-infection in an endemic area in Northeast Brazil. Rev Soc Bras Med Trop. 2021;54:e0795. doi: 10.1590/0037-8682-0795-2020 33886819 PMC8047714

[32] Pereira DA, Almeida MT, Lacerda RB, Santos JA. Temporal analysis of human visceral leishmaniasis (HVL) notifications in the Microregion of Pirapora-MG. Rev Cient Esc Estadual Saude Publica Cândido Santiago. 2023;9(9c5):1–20. doi: 10.22491/2447-3405.2023.V9.9c5

[33] de Sousa Félix de Lima M, Albuquerque E Silva R, de Almeida Rocha D, de Oliveira Mosqueira G, Gurgel-Gonçalves R, Takashi Obara M. Insecticide-impregnated dog collars for the control of visceral leishmaniasis: evaluation of the susceptibility of field *Lutzomyia longipalpis* populations to deltamethrin. Parasit Vectors. 2024;17(1):468. doi: 10.1186/s13071-024-06474-4 39548568 PMC11566624

[34] Dembelu M, Woseneleh T. Prevalence of and factors associated with reoccurrence of opportunistic infections among adult HIV/AIDS patients attending the ART clinic at public health facilities in Arba Minch Town, Southern Ethiopia. HIV AIDS (Auckl). 2021;13:867–76. doi: 10.2147/HIV.S328362 34512035 PMC8427687

[35] Werneck GL, Figueiredo FB, Cruz M do SPE. Impact of 4% deltamethrin-impregnated dog collars on the incidence of human visceral Leishmaniasis: a community intervention trial in Brazil. Pathogens. 2024;13(2):135. doi: 10.3390/pathogens13020135 38392873 PMC10892744

[36] Vergara MPP. LV-HIV/AIDS coinfection: challenges in patient treatment and management [Master’s dissertation]. São Paulo: Universidade de São Paulo; 2014. doi: 10.11606/D.5.2005.tde-10102014-110912

[37] Lindoso JAL, Cunha MA, Queiroz IT, Moreira CHV. Leishmaniasis-HIV coinfection: current challenges. HIV AIDS (Auckl). 2016;8:147–56. doi: 10.2147/HIV.S93789 27785103 PMC5063600

[38] Monge-Maillo B, Norman FF, Cruz I, Alvar J, López-Vélez R. Visceral leishmaniasis and HIV coinfection in the Mediterranean region. PLoS Negl Trop Dis. 2014;8(8):e3021. doi: 10.1371/journal.pntd.0003021 25144380 PMC4140663

[39] Stefani Cesar Lima R, Ramalho Oliveira M, Alves de Albuquerque B, Nunes da Conceição H, Mourão Pereira B, Da Costa Ximenes J, et al. Perfil clínico, epidemiológico e espacial de leishmaniose visceral em área endêmica do estado do Maranhão, Brasil. Mundo Saude. 2020;44:171–82. doi: 10.15343/0104-7809.202044171182

[40] Monteiro BEF, da Silva ED, Bezerra GSN, Cavalcante MK de A, Pereira VRA, Castro MCAB, et al. Evaluation of proinflammatory chemokines in HIV patients with asymptomatic *Leishmania infantum* infection. Trop Med Infect Dis. 2023;8(11):495. doi: 10.3390/tropicalmed8110495 37999614 PMC10675805

[41] Cunha MA, Celeste BJ, Kesper N, Fugimori M, Lago MM, Ibanes AS, et al. Frequency of *Leishmania* spp. infection among HIV-infected patients living in an urban area in Brazil: a cross-sectional study. BMC Infect Dis. 2020;20(1):885. doi: 10.1186/s12879-020-05622-2 33238943 PMC7686951

[42] Queiroz MJA, Alves JGB, Correia JB. Leishmaniose visceral: características clínico-epidemiológicas em crianças de área endêmica. J Pediatr (Rio J). 2004;80(2):141–6. doi: 10.1590/s0021-75572004000200012 15079185

